# Genome Sequences and Characteristics of Six Cluster B1 Mycobacteriophages Discovered at Saint Joseph’s University

**DOI:** 10.1128/mra.00754-22

**Published:** 2022-09-22

**Authors:** Anne Winkler, April Pivonka, Aidan Conry-Murray, Cecilia Petruconis, Isabella Patterne, Bernadette Bergman, Elizabeth Binder, Joshua Blackley, Rachel Brown, Katherine Commale, Emily Costello, Taylor Cromer, Jasmine Davila, Olivia DeSanto, Mary Agnus Dunn, Deborah Duong, Sophia Feingold, Kayla Flanders, Mary Frattara, Tate Fryczynski, Leya Givvines, Dana Glavin, Reid Hartman, Julia Iacovella, Katherine Koestler, Caroline Kominick, Andy Lam, Sharon Mashkovich, Jordan McCarthy, Corinne Merlino, Alexa Mihaita, Kara Moulton, Thientrinh Nguyen, Danielle Niblock, Isabella Paoli, Skye Rodriguez, Isabella Stefanic, Jenna Stoneroad, Caren Teague, Fabiana Tort-Umpierre, Arianna Varano, Alexandra Vlahovic, John Braverman, Christina King-Smith, Julia Y. Lee-Soety

**Affiliations:** a Department of Biology, Saint Joseph’s University, Philadelphia, Pennsylvania, USA; DOE Joint Genome Institute

## Abstract

We report on six new siphoviruses infecting Mycobacterium smegmatis that were isolated from soil samples collected on the campus of Saint Joseph’s University, on the western edge of Philadelphia, Pennsylvania. All phages have circularly permuted genomes that are 68,721 to 68,929 bp long, with an average G+C content of 66.4%.

## ANNOUNCEMENT

**FIG 1:**
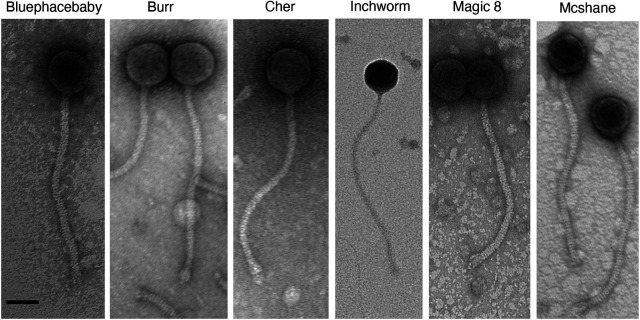
Six phages in the B1 cluster show *Siphoviridae* morphology. Phage lysates were stained with 1% uranyl acetate. Scale bar, 50 nm.

The clinical significance of bacteriophages and the need to discover additional phages are highlighted in recent reports describing the treatment of multidrug-resistant Mycobacterium abscessus infections using a mycobacteriophage cocktail (1–3). From soil samples collected by Saint Joseph’s University students (Table 1), we isolated six bacteriophages that infect Mycobacterium smegmatis mc^2^155, using standard methods (3). Briefly, soil samples were resuspended in 7H9 liquid medium and filtered (0.2-μm pore size). Filtrates from two soil samples were plated in top agar with M. smegmatis, yielding phages Inchworm and Magic8. The remaining four filtrates were inoculated with M. smegmatis, incubated with shaking for 2 days at 37°C, filtered, and plated in top agar with M. smegmatis, which yielded phages Bluephacebaby, Burr, Cher, and Mcshane. All phages were purified with at least three rounds of plating; all formed clear plaques (3 to 4 mm in diameter) after 24 to 48 h at 37°C. Negatively stained transmission electron micrographs revealed all phages to be similarly sized siphoviruses, with icosahedral capsids (66.9 ± 6.8 nm [*N *= 27]) and long noncontractile tails (309.8 ± 14.9 nm [*N *= 27]) (Fig. 1).

**TABLE 1:** Sample collection information, DNA isolation method, sequencing results, and genome characteristics for six mycobacteriophages in the B1 cluster

Phage name (mo/yr of sample collection)	GenBank accession no.	SRA accession no.	Sample collection location (GPS coordinates)	DNA isolation method	No. of reads (×1,000)	Approx coverage (×)	Genome length (bp)	G+C content (%)	No. of genes
Bluephacebaby (10/2019)	MW534381	SRX11422989	39.993965, −75.238488	Wizard DNA kit	455.2	935	68,899	66.5	101
Burr (9/2018)	MW712720	SRX11422993	39.994400, −75.240500	Phenol-chloroform	719.2	1,480	68,721	66.5	104
Cher (9/2018)	MW712726	SRX11422994	39.993800, −75.238900	Phenol-chloroform	721.1	1,482	68,855	66.4	102
Inchworm (11/2019)	MT889369	SRX11422999	39.994100, −75.237900	Wizard DNA kit	1,100	2,173	68,929	66.3	103
Magic8 (10/2019)	MT776813	SRX11423000	39.996000, −75.231000	Wizard DNA kit	762.1	1,559	68,865	66.4	96
Mcshane (9/2018)	MN703415	SRX11422990	39.993810, −75.238795	Phenol-chloroform	476.7	976	68,929	66.4	104

Phage DNA was isolated with the Promega Wizard DNA cleanup kit (3) or phenol-chloroform extraction (https://phagesdb.org/protocols/88), prepared for sequencing with the NEBNext Ultra II DNA library preparation kit, and sequenced on an Illumina MiSeq system (v3 reagents), which yielded 455.2 thousand to 1.1 million single-end 150-base reads. Sequences were assembled and checked for completeness using Newbler (v2.9) and Consed (v29) (4), respectively, generating single major contigs with 935- to 2,173-fold coverage. Multiple DNA sequence alignment (Geneious v10.2.2) showed these six phage genomes to be highly similar to one another, sharing over 95% nucleotide identity. All phages were assigned to cluster B1 based on clustering parameters of at least 35% shared protein homologs (phams) with other cluster B1 phages in the Actinobacteriophage Database (5). All of the genomes are circularly permuted, with G+C contents of 66.3 to 66.5% (Table 1).

Phage genomes were annotated using PECAAN (v2019-20) (6) or DNA Master (v5.23.2) (7), Phamerator (v477) (8), Starterator (v1.0.1 and v1.2) (http://phages.wustl.edu/starterator), ARAGORN (v2) and tRNAscan-SE (v1.2.38) (9, 10), and HHPred (v3.2, with PDB_mmCIF70, SCOPe70, Pfam-A, and NCBI conserved domain [CD] databases) and BLASTP (v2.9, with Actinobacteriophage Database protein and NCBI nonredundant protein sequence databases) (11, 12). All bioinformatic tools were used with default parameters. An average of 102 genes were identified in each genome, with no tRNA/transfer-messenger RNA genes.

The six genomes share similar functional organizations, with structure and assembly gene homologs, including terminase, portal protein, capsid maturation protease and MuF-like fusion protein, major capsid protein, head-to-tail adaptor, tail assembly chaperone, tape measure protein, and minor tail proteins, being encoded on the left half of the genome. Each genome also encodes HNH endonucleases, which are often associated with terminase for DNA packaging (10). Scaffolding proteins typically associated with capsid maturation proteases were not identified (13). All six phages also contain a gene encoding DpdA-like tRNA-guanine transglycosylase, but no other genes in the preQ_0_ pathway, required to modify specific guanine residues for protection from host restriction enzymes, could be identified (14). These six new B1 members bring the total number of phages in this cluster to 239.

### Data availability.

See Table 1 for the accession numbers for all six phages.

## References

[1] Nick JA, Dedrick RM, Gray AL, Vladar EK, Smith BE, Freeman KG, Malcolm KC, Epperson LE, Hasan NA, Hendrix J, Callahan K, Walton K, Vestal B, Wheeler E, Rysavy NM, Poch K, Caceres S, Lovell VK, Hisert KB, de Moura VC, Chatterjee D, De P, Weakly N, Martiniano SL, Lynch DA, Daley CL, Strong M, Jia F, Hatfull GF, Davidson RM. 2022. Host and pathogen response to bacteriophage engineered against *Mycobacterium abscessus* lung infection. Cell 185:1860–1874.e12. doi:10.1016/j.cell.2022.04.024. 35568033 PMC9840467

[2] Dedrick RM, Guerrero-Bustamante CA, Garlena RA, Russell DA, Ford K, Harris K, Gilmour KC, Soothill J, Jacobs-Sera D, Schooley RT, Hatfull GF, Spencer H. 2019. Engineered bacteriophages for treatment of a patient with a disseminated drug-resistant *Mycobacterium abscessus*. Nat Med 25:730–733. doi:10.1038/s41591-019-0437-z. 31068712 PMC6557439

[3] Dedrick RM, Smith BE, Cristinziano M, Freeman KG, Jacobs-Sera D, Belessis Y, Whitney Brown A, Cohen KA, Davidson RM, van Duin D, Gainey A, Garcia CB, George CRR, Haidar G, Ip W, Iredell J, Khatami A, Little JS, Malmivaara K, McMullan BJ, Michalik DE, Moscatelli A, Nick JA, Tupayachi Ortiz MG, Polenakovik HM, Robinson PD, Skurnik M, Solomon DA, Soothill J, Spencer H, Wark P, Worth A, Schooley RT, Benson CA, Hatfull GF. 2022. Phage therapy of *Mycobacterium* infections: compassionate-use of phages in twenty patients with drug-resistant mycobacterial disease. Clin Infect Dis ciac453. doi:10.1093/cid/ciac453. 35676823 PMC9825826

[4] Russell DA. 2018. Sequencing, assembling, and finishing complete bacteriophage genomes. Methods Mol Biol 1681:109–125. doi:10.1007/978-1-4939-7343-9_9. 29134591

[5] Pope WH, Mavrich TN, Garlena RA, Guerrero-Bustamante CA, Jacobs-Sera D, Montgomery MT, Russell DA, Warner MH, Science Education Alliance-Phage Hunters Advancing Genomics and Evolutionary Science, Hatfull GF. 2017. Bacteriophages of *Gordonia* spp. display a spectrum of diversity and genetic relationships. mBio 8:e01069-17. doi:10.1128/mBio.01069-17. 28811342 PMC5559632

[6] Rinehart CAGB, Smith JR, Wood JD. 2016. PECAAN: Phage Evidence Collection and Annotation Network user guide. Western Kentucky University Bioinformatics and Information Science Center, Bowling Green, KY https://seaphages.org/media/docs/PECAAN_User_Guide_Dec7_2016.pdf.

[7] Pope WH, Jacobs-Sera D. 2018. Annotation of bacteriophage genome sequences using DNA Master: an overview. Methods Mol Biol 1681:217–229. doi:10.1007/978-1-4939-7343-9_16. 29134598

[8] Cresawn SG, Bogel M, Day N, Jacobs-Sera D, Hendrix RW, Hatfull GF. 2011. Phamerator: a bioinformatic tool for comparative bacteriophage genomics. BMC Bioinformatics 12:395. doi:10.1186/1471-2105-12-395. 21991981 PMC3233612

[9] Schattner P, Brooks AN, Lowe TM. 2005. The tRNAscan-SE, snoscan and snoGPS web servers for the detection of tRNAs and snoRNAs. Nucleic Acids Res 33:W686–W689. doi:10.1093/nar/gki366. 15980563 PMC1160127

[10] Chan PP, Lowe TM. 2019. tRNAscan-SE: searching for tRNA genes in genomic sequences. Methods Mol Biol 1962:1–14. doi:10.1007/978-1-4939-9173-0_1. 31020551 PMC6768409

[11] Altschul SF, Gish W, Miller W, Myers EW, Lipman DJ. 1990. Basic local alignment search tool. J Mol Biol 215:403–410. doi:10.1016/S0022-2836(05)80360-2. 2231712

[12] Soding J, Biegert A, Lupas AN. 2005. The HHpred interactive server for protein homology detection and structure prediction. Nucleic Acids Res 33:W244–W248. doi:10.1093/nar/gki408. 15980461 PMC1160169

[13] Duda RL, Oh B, Hendrix RW. 2013. Functional domains of the HK97 capsid maturation protease and the mechanisms of protein encapsidation. J Mol Biol 425:2765–2781. doi:10.1016/j.jmb.2013.05.002. 23688818 PMC3709472

[14] Hutinet G, Kot W, Cui L, Hillebrand R, Balamkundu S, Gnanakalai S, Neelakandan R, Carstens AB, Fa Lui C, Tremblay D, Jacobs-Sera D, Sassanfar M, Lee YJ, Weigele P, Moineau S, Hatfull GF, Dedon PC, Hansen LH, de Crecy-Lagard V. 2019. 7-Deazaguanine modifications protect phage DNA from host restriction systems. Nat Commun 10:5442. doi:10.1038/s41467-019-13384-y. 31784519 PMC6884629

